# Effect of Deep Placement Fertilization on the Distribution of Biomass, Nutrients, and Root System Development in Potato Plants

**DOI:** 10.3390/plants12091880

**Published:** 2023-05-04

**Authors:** Tomasz Niedziński, Beata Rutkowska, Jan Łabętowicz, Wiesław Szulc

**Affiliations:** Independent Department of Agricultural and Environmental Chemistry, Institute of Agriculture, Warsaw University of Life Sciences, Nowosynowska 166, 02-787 Warsaw, Poland

**Keywords:** deep fertilization, biomass distribution, nutrient distribution, potato root system

## Abstract

The study was carried out in designed pots—rhizoboxes. Root systems were evaluated using computer scanning to determine total length, root area, and root diameter. The study showed a favorable effect of deep placement of fertilizers on total yield, increasing biomass yield by 7–17% relative to surface fertilization. The largest biomass increase under the influence of deep fertilization was obtained in the case of tuber yield, in which a yield increase of 18–34% was obtained. Higher yields of potato tubers were obtained under depth fertilization compared to surface application of fertilizers. Under the influence of deep fertilization at a depth of 20 cm, the uptake of nitrogen and phosphorus by potato biomass increased by 20–21%. Increased depth of fertilization increased the proportion of nitrogen accumulated in the tubers, while in the case of phosphorus, no effect of depth on P distribution was shown. An analysis of root system parameters showed a positive effect on increases in length and total root area under deep fertilization of potato plants. Based on the study, it was found that the distribution of dry matter, nutrients, and potato root development parameters were most optimal when fertilizer granules were applied at a depth of 20 cm.

## 1. Introduction

The distribution of fertilizer granules in the rhizosphere of a developing root system contributes to the modification of its morphology [1]. Plant roots develop in the soil in three dimensions [2], and root architecture largely depends on genetic factors [3]. The variable properties of the growth environment force changes in the architecture of the root system, ensuring effective uptake of nutrients and water [4,5,6]. Under conditions of nutrient deficit, the growth of the main root is limited in favor of growth of lateral roots, development of root hairs, and increased density of root growth in a unit of soil [7,8,9]. Lateral roots constitute 90–98% of the total length of roots and account for the total length of roots to the greatest extent [10,11]. This development strategy involves an increase in range aimed at meeting water and nutrient needs [5]. Root morphology changes under the influence of nutrients. Higher concentrations of nitrate nitrogen NO_3_^−^ > 10 mM stimulate the growth of existing lateral roots, whereas the presence of ammonium ions strengthens the intensity of the development of branches of higher order roots [12]. When the source of nitrogen is amino acids, the root system reaches higher development in comparison to inorganic nitrogen sources [13]. High availability of nutrients, particularly where granules are placed, increases the intensity of growth of lateral roots [14]. Growth of the root system is of compensatory character. Roots thrive in zones with abundant available forms of nitrogen and phosphorus. The growth is compensated and can be proportionate to the development of roots in zones that are poor in nitrogen and phosphorus [15]. Compensation growth contributes to the stimulation of the development of root hairs in zones that are poor in the nutrients responsible for strengthening the aggregate structure of soil [16]. Nutrients are absorbed the most intensively by the youngest root zone, directly behind the meristem [17,18]. Phosphorus deficits change root architecture, limiting the development of the main root in favor of lateral roots [19,20]. Phosphorus concentration in the soil solution often reaches values at a level from 0.1 to 10 µM, providing no possibility of meeting the nutritional needs of plants [21]. Phosphorus supply to plants is limited by a low diffusion coefficient, ranging from 10−12 to 10−15 m^2^∙s^−1^ [22]. The capacity to absorb phosphorus from soil depends on the accompanying ions in the soil solution [23]. The presence of ions in the soil solution, both NH_4_^+^ and H_2_PO_4_^2−^, causes an increase in the efficiency of uptake of both these components, an increase in the growth of lateral roots [24], and an increase in local soil acidification [25]. Intensification of rooting increases the total surface area of roots, thereby enhancing the potential for efficient uptake of phosphorus from soil [26]. By limiting contact of fertilizer with soil, row application of phosphorus and ammonia nitrogen ensures local concentration of both elements in the application zone, thereby contributing to an increase in the length and density of roots [25]. Row fertilization with ammonium ions and phosphorus stimulates an increase in the length and density of roots during the application of fertilizer granules down into the soil in the 0–30 cm layer compared to surface fertilization [25]. High root density in the layer up to 50 cm limits the washing out of nutrients by infiltration water. In the surface layer, lateral roots reach a high share [27], and with depth, the share decreases [28,29]. Roots with larger than average diameters have no ability to proliferate [30]. Due to the higher number of bark cells, they require greater supply of C to maintain respiration. Moreover, limited possibilities of nutrient uptake reduce the efficiency of C supply of the above-ground parts to the roots. This can have a negative effect on the production of the biomass of the above-ground part, which consequently causes a decrease in yield [31]. Deposit fertilization with ammonium nitrogen protects plant roots against the harmful effects of the ammonium form. Nitrogen uptake is controlled by the development of plants with consideration of the current nutritional needs and growth intensity (CULTAN—controlled uptake long term ammonium nutrition) [32]. Correct root development around the deposit, presence of root apices, and appropriate supply of carbohydrates to roots permit nitrogen uptake. In such conditions, root apices located near the ammonium deposit are supplied with carbohydrates earlier than other parts of the plant, promoting the development of the root system against the development of the above-ground parts.

This paper presents the results of the effects of different depths of fertilizer placement on yield, dry matter distribution, nutrient distribution in the plant, and development of the potato root system.

## 2. Results and Discussion

### 2.1. Distribution of Biomass to Above-Ground Parts, Roots, and Tubers

Fertilization considerably increased the total yield of plant biomass compared to objects with no fertilization (Table 1). The total plant biomass in the object with no fertilization (D_0_) was approximately 43 g. On fertilized objects, the total biomass increased from approximately 77 to 88 g d.m. per plant. Nitrogen fertilization increases the total yield of potato biomass [33], usually investigated in a range of nitrogen fertilization of up to 330 kg∙ha^−1^ [34]. An increase in potato yield is primarily related to the application of N. Phosphorus (P) and potassium (K) affect potato yielding [35]. The availability of nitrogen in soil determines the development of the yield and accumulation of biomass both in the above-ground and underground parts [36]. A considerable effect of the depth of placing granules on total potato yield was evidenced. The conducted experiment revealed that the optimal depth for placing fertilizer granules was at a level of 20 cm for all levels of fertilization. In all deep fertilization variants, a biomass yield 7–17% higher compared to surface application was obtained. The positive effect of deep mineral or organic fertilization on potato yielding has also been evidenced in other studies, particularly at a depth of 25–35 cm [37]. Next to nitrogen, applying phosphorus and potassium as well can increase the potato yield by as much as 16% [38].

The biomass yield of the above-ground parts of the potato plants showed strong dependency on the accumulation of assimilated substances in the above-ground parts on fertilization (Table 2). The yield of the above-ground parts on objects with no fertilization was 7.7 g per plant, which increased on average more than three times as a result of fertilization, reaching a yield from 20 g to 39 g per plant. In study [39], nitrogen fertilization of 2.5, 8.0, and 16.0 g N∙plant^−1^ strongly contributed to growth of the above-ground parts. High doses of 8.0 and 16.0 g N∙plant^−1^ caused four-fold higher growth of the above-ground parts in comparison to a dose of 2.5 g N∙plant^−1^.

In study [40], phosphorus fertilization contributed more to increases in the yield of tubers than the above-ground parts. The accumulation of the yield of dry mass of the above-ground parts was determined less by the variable depth of the placement of fertilizer granules. The maximum yield of the dry mass of above-ground parts occurred in the case of applying granules at a depth of 10 cm.

The yielding of tubers depends on nitrogen fertilization. An increase in available nitrogen causing an increase in the yield of tubers is usually observed in a range of 0–300 kg N∙ha^−1^. In study [41], an increase in yield as a result of N fertilization from 36 to 49 g increased the share of tuber biomass in total biomass to 76–87% [42]. This study evidenced that the accumulation of biomass in potato tubers showed a course that was different from that in the case of above-ground parts and roots. A decrease in the yield of tubers was observed as a result of an increase in fertilizer dose. The fertilizer dose D_1_ proved optimal for the yield of tubers. The results of research on the effect of fertilization level show that exceeding the optimum level of nitrogen fertilization [43,44] for specific conditions contributes to a decrease in tuber yielding, even at a level of more than 160 kg N∙ha^−1^. The effect is dependent on the development stage of the potato [35]. In research by Kavvadias et al. [45], an increase in the N dose from 330 to 495 kg N∙ha^−1^ caused a decrease in yielding and harvest index value. In the same study, the authors showed no effect of potassium fertilization in a range from 112 to 450 kg K_2_O∙ha^−1^ but only revealed significant growth of tubers per plant.

The yielding of potato tubers was largely dependent on the depth of the application of granules (Table 3). The highest yield of tubers was obtained in the case of placing the granules at a depth of 20 cm. Deep application permitted obtaining a yield higher by 18–34% in comparison to the yield obtained on the object with fertilization on the soil surface. In study [37], the application of deep fertilization caused an increase in the yield of tubers. In comparison to surface fertilization, the optimum depth of fertilization was 15 cm, which increased the yield of tubers by 17%. A further increase in the fertilization depth to 25 cm caused a 9% decrease in the yield in comparison to surface fertilization. The application of deep fertilization with phosphorus and potassium in study [38] allowed for an increase in the yield of tubers by 16%. In study [37], fertilization at a depth from 25 up to 35 cm provided the best yield-forming results. The yield of tubers in comparison to surface fertilization increased significantly from 39.1 t∙ha^−1^ to 51.2 t∙ha^−1^. Higher yielding also occurred in the case of fertilization at a depth of 15 cm in comparison to surface fertilization. An increase in fertilization depth to 25 cm caused a decrease in the yield.

With an increase in fertilization, a proportionate increase in root mass was observed. Between the lowest (D_1_) and highest level of fertilization (D_3_), an approximately two-fold increase in root mass occurred (Table 4). The obtained results correspond with those of other authors, evidencing that an increase in nitrogen fertilization caused an increase in the accumulation of the dry mass of roots [42]. Roots developed the highest amount of biomass at a depth of application of 10 cm, which was 10% higher in comparison to surface fertilization.

Deeper placement of fertilizers reduced the growth of root mass in comparison to surface application by 5–20%. In study [37], mineral fertilization with NPK with surface application resulted in a yield of root biomass of 0.63 g d.m.∙plant^−1^. Fertilization at a depth of 15, 25, and 25–35 cm caused an accumulation of root biomass of 0.41, 0.55, and 0.74 g d.m.∙plant^−1^, respectively.

### 2.2. Uptake and Distribution of Nitrogen and Phosphorus

Uptake of nitrogen and phosphorus is correlated with the dose of fertilizer applied and the method of its application [46]. Few papers have discussed the effects of the depth of placing fertilizers on the uptake of nutrients by potato plants. Various methods of fertilizer application, including deep application, increased the yield in different species by 3.7%, the content of nutrients by 3.7%, and the uptake of nutrients by the above-ground parts by an average of 11.9% compared to surface fertilization. A positive effect on the yield and uptake generally occurs at a depth of fertilization of at least 10 cm [47]. After the application of deep row fertilization, Westermann and Sojka [48] obtained an uptake of nitrogen and phosphorus by potatoes that was higher by 28 and 11.6%, respectively. Our own research confirmed the positive effect of deep application of nitrogen and phosphorus on their uptake by potato biomass. The highest amount of nutrients was absorbed when fertilizer was applied at a depth of 20 cm. In comparison to surface fertilization, an increase in nitrogen and phosphorus uptake reached 21 and 20%, respectively. Phosphorus uptake doubled due to fertilization. Its uptake in comparison to nitrogen was approximately 10 times lower (Table 5). The distribution of nitrogen and phosphorus in the plants was variable, both in terms of dose and fertilization depth. The greatest amount of elements accumulated in the biomass of the above-ground parts and in the tubers. The roots constituted only a small share of the uptake of nutrients, accounting for a maximum of 10–13% of the total amount.

**Figure 1:**
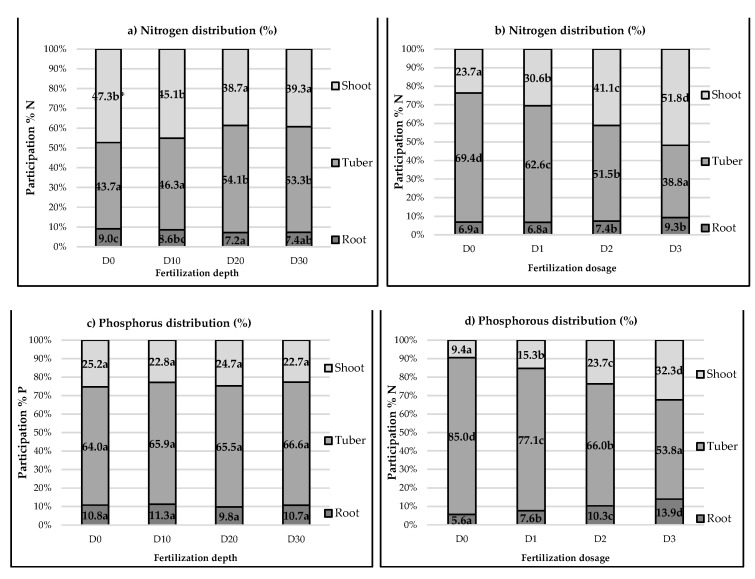
Effect of fertilization depth on the distribution of nitrogen (**a**) and phosphorus (**c**) and effect of fertilization rate on the distribution of nitrogen (**b**) and phosphorus (**d**) in total biomass. * Average values of the proportion of N and P uptake for different depths and fertilization dosages, denoted by the same letter, were not significantly differentiated at the level of α = 0.05.

The depth of placing fertilizers had the effect of changing the distribution of absorbed nitrogen, whereas no significant changes were recorded for phosphorus. An increase in depth reduced the share of nitrogen accumulated in the above-ground parts, from 47% to 39%, in favor of accumulation in the tubers, increasing the share from 43% to 53%. An increase in the level of fertilization was of high importance in the distribution of both nitrogen and phosphorus. An increase in the fertilizer dose caused an increase in the share of absorbed nitrogen and phosphorus in the above-ground parts and resulted in a decrease in the share of the tubers in total nutrient uptake (Figure 1). The obtained results correspond with the conclusion of another study [47] that determined a positive effect of fertilization at a depth of >10 cm on an increase in the accumulation and share of nitrogen in the above-ground parts of various plant species, particularly when phosphorus was applied together with nitrogen.

Fertilization with nitrogen and phosphorus, with maintenance of a constant proportion of the elements in fertilization, causes considerably faster uptake of nitrogen from soil by the root system of plants than phosphorus (Table 6). Nutrients differ in their mobility (effective diffusion coefficient) and rate of uptake by the root system [49]. Both urea (0.62∙10^−6^ cm^2^∙s^−1^) and ammonium ions (0.157∙10^−6^ cm^2^∙s^−1^) show higher mobility in soil [50,51] in comparison to phosphate ions (0.00001–0.01∙10^−6^ cm^2^∙s^−1^) [49].

Due to fertilization, the dynamics of nitrogen accumulation toward phosphorus were more than two times higher. The experiment showed that with an increase in fertilizer dose, the weight ratio of the absorbed fertilizer increased toward phosphorus. The rate of nutrient uptake was affected by fertilization depth. In comparison to surface fertilization, only fertilization at a depth of 10 cm increased the proportion of absorbed N:P. A further increase in fertilization depth reduced the uptake ratio of N:P.

### 2.3. Root System Development

**Figure 2:**
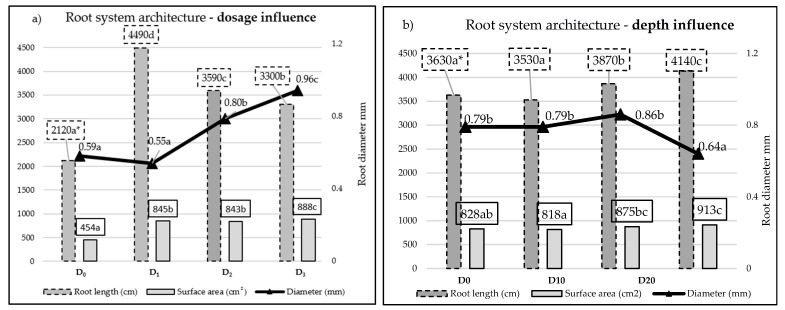
Effect of fertilization rate (**a**) and fertilization depth (**b**) on mean values of length, total area, and diameter of the root system. * Average root values for different fertilization rates/depths, denoted by the same letter, were not significantly differentiated at a level of α = 0.05.

The root system of potato plants primarily develops in the surface soil layer of up to 30 cm. Only a small share of roots reaches a length of more than 1 m [52]. In the period of initial growth of 3–5 weeks, they develop at a rate of 1.2 cm∙day^−1^. The second period of slower growth (approximately 0.8 cm per day) was followed by the discontinuation of elongation of roots until the end of life of the plant. The length of roots is dependent on both the potato cultivar and fertilization. It can reach a value from 1.1 to 1.7 km∙m^−2^ [53]. The distribution of roots in the seed row in the absence of conditions disturbing the growth is uniform up to a depth of 50 cm, and the highest root density of 5.5 cm∙cm^−3^ occurs in the zone of 20 cm depth. The development of potato roots is stimulated by the occurrence of a factor limiting growth, e.g., water, when the plant produces a stronger, more developed root system [52]. In the study, for objects subject to fertilization, the length of roots increased more than two times in comparison to the control. An increase in the dose from D_1_ to D_3_ caused a decrease in the length of roots from 4490 cm∙plant^−1^ to 3300 cm∙plant^−1^ (Figure 2).

**Figure 3:**
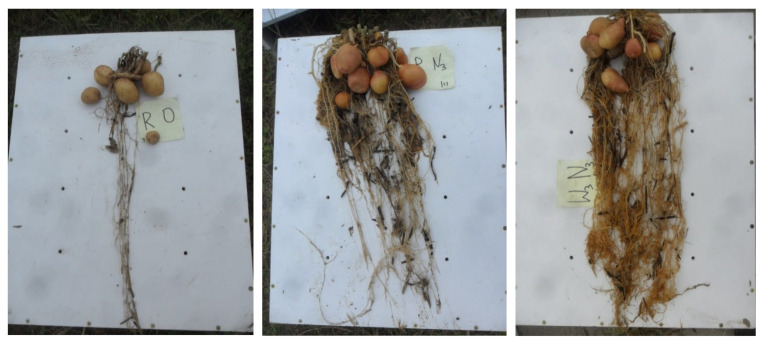
Potato root system size—experiment in the greenhouse, from left: D_0_ (without fertilization_)_; deep placement: D10 and D30.

Deep fertilizer application significantly increased the length of the root system, reaching a maximum length (4490 cm) at the deepest placement of fertilizers—at a depth of 30 cm, which was 14% higher in comparison to the object with surface fertilizer application (Figure 3).

Fertilization strongly contributed to an increase in the surface area of the root system from 454 cm^2^ ·plant^−1^ to as much as 888 cm^2^ ·plant^−1^ for the control object and dose of D_3_, respectively. Deep application increased the total surface area of the roots in comparison to surface fertilizer application. The highest surface area of 913 cm^2^ ·plant^−1^ was reached on the object with fertilization at a depth of 30 cm, which was higher by 10% in comparison to the object with surface application.

The analysis of root diameters showed morphological changes expressed in the growth of the root diameter. An abundant supply of nutrients to plants reduces the share of small roots with a smaller diameter in favor of thicker roots in the root system. The effect of fertilization depth was evidenced. Up to a depth of fertilization of 20 cm, the roots showed no variability in terms of diameter. In the case of an increase in fertilization depth, a decrease in root diameter was shown.

## 3. Materials and Methods

**Figure 4:**
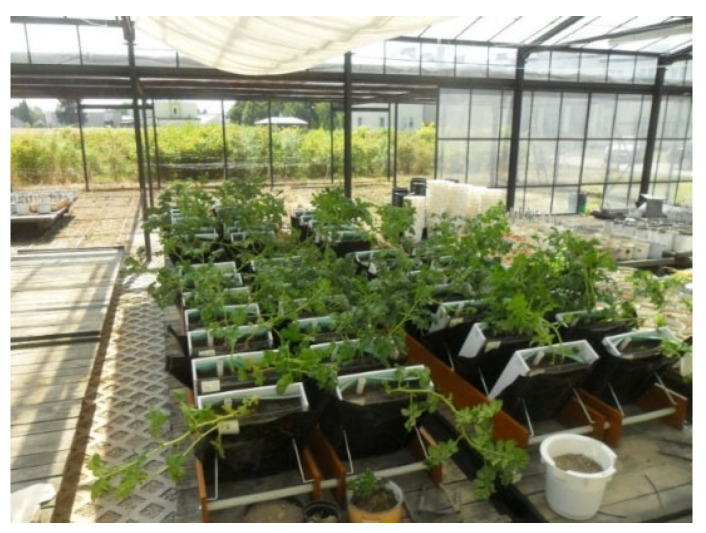
Pot experiment in rhizoboxes.

The pot experiment in a rhizobox system was conducted at the Experimental Station of the Faculty of Agriculture and Ecology of the Warsaw University of Life Sciences in Skierniewice over a period of two years. The pots with a cuboid shape with dimensions of 40 × 15 × 50 cm were made of PVC material with the possibility of visual observation of root growth (Figure 4).

The experiment was established using soil from an arable field surface layer Ap of podzolic soil belonging to the granulometric group of light loamy sand (Table 7). The pots were filled with soil to the amount of 22 kg each. Constant moisture at a level of 60% of maximum water capacity was maintained during vegetation. Soil reaction was measured potentiometrically in 1 M KCl (ISO 10390:2022-09), soil abundance in available forms of phosphorus and potassium by means of the Egner-Riehm method (ER_DL_) (PN-R-04023:1996, PN-R-04022:1996), and magnesium by means of the Schachtschabel method (PN-R-04020:1996).

### 3.1. Experimental Design

**Figure 5:**
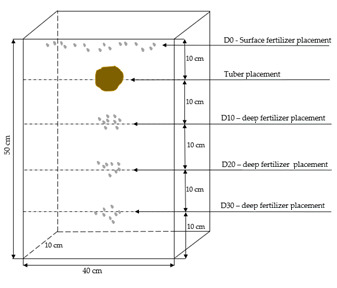
Scheme of fertilization.

Tubers were placed at a depth of 10 cm, one tuber per pot, and fertilizer granules were applied at various depths: D0 (surface fertilization), D10 (fertilization at a depth of 10 cm), D20 (at a depth of 20 cm), and D30 (at a depth of 30 cm) (Figure 5). Tubers were selected of equal weight and were stimulated and germinated before planting.

Four levels of fertilization were applied: D_0_, D_1_, D_2_, and D_3_, corresponding to the doses of fertilizer elements presented in Table 8.

### 3.2. Fertilizer Study

Fertilization employed the fertilizer UreaPhoS-Micro, with a granule diameter increased to 10 mm. The fertilizer had the following content of elements: nitrogen (N)—200, phosphorus (P_2_O_5_)—100, sulphur (S)—70, copper (Cu)—1.5, zinc (Zn)—3.0, and boron (B)—0.6 g kg^−1^. The fertilizer contained urea adduct combined with calcium sulphide with a hydrogen bond, resulting in calcium tetra urea CaSO_4_·4CO(NH_2_)_2_, according to the following formula (Malinowski et al. 2007): CaSO_4_·nH_2_O + 4CO(NH_2_)_2_ → CaSO_4_·4CO(NH_2_)_2_. Potassium was applied in the form of 60% potassium salt. The experiment employed a medium early cultivar Irga. The plant material was collected in the phase of intensive growth of tubers, weighed, and dried in natural conditions to obtain air-dry mass. The dry mass was subject to the determination of total content of nitrogen after Kjeldahl (ISO 1871:2009) and phosphorus by means of the spectrophotometric method with ammonium metavanadate. Nutrient uptake was calculated based on the following formula:

(1) A=Y·U

where A—uptake of element in g∙plant^−1^, Y—yield of dry mass (g), and U—content of element in dry mass.

Yield of dry mass and uptake of nutrients were expressed in relative numbers, comparing the obtained values with combinations with surface fertilization D0.

(2) $$\mathrm{RY},\;\mathrm{RU}=\frac{Yo,\;\;\;Uo}{Yd,\;\;\;Ud}\;\cdot 100\%$$

where RY—relative yield; *Yo*, *Uo*—yield or uptake on combination with surface fertilization D0; *Yd*, *Ud*—yield or uptake on combination with fertilization at a depth of D10, D20, or D30.

**Figure 6:**
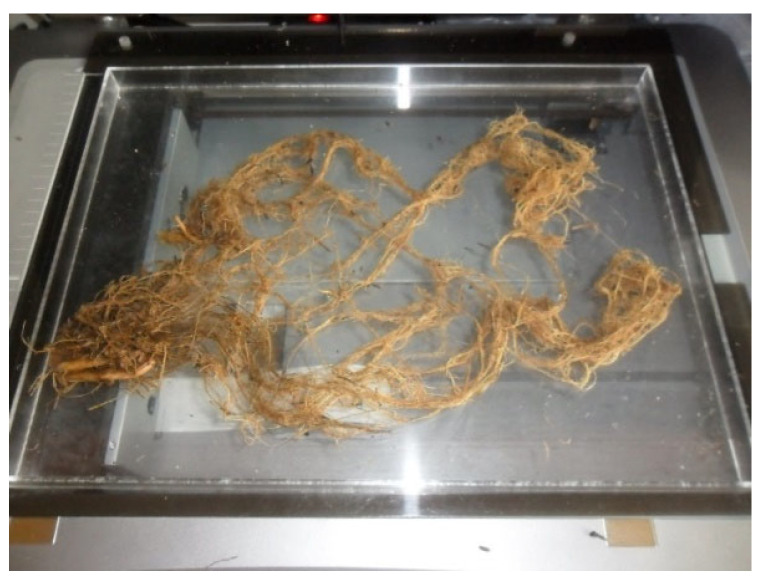
Root system scanner EPSON EXPRESSION 10000XL.

The parameters of root system architecture, namely total length (m), total surface area (cm^2^), and root diameter (mm), were determined by means of a root scanner EPSON EXPRESSION 10000XL (Figure 6) with WinRhizo software after previous water extraction of the roots from the soil. The root system was taken 90 days after planting equally over a two-year period, at the physiological activity stage, when the above-ground parts were still green. The yields of the aboveground parts, tubers, and roots were determined, and root system parameters were determined using fresh root analysis in the root scanner. Subsequently, all plants were then dried to air-dry weight, the samples were homogenized, each part was very finely ground, and the nutrient contents of each plant and each part of the plant were determined. Increasing mean values were marked with consecutive letters.

Total nitrogen content in the above-ground and underground parts was determined in accordance with the Kjeldahl procedure. Phosphorus was determined spectrophotometrically with vanadium ammonium molybdate.

### 3.3. Statistical Analysis

The experiment was established in a random system. The collected data from two years, done in four replicates, with eight replicates (n = 8) per treatment, were processed for the effect of fertilizer placement and application rate on growth and nutrient uptake. The data were collected from 128 pots: 4 fertilizer placements × 4 fertilizer dosages × 8 replications; above-ground parts, tubers, and roots were separated, and statistical analysis was performed for each plant part and for whole plants. Analysis of variance was performed for the means obtained from the treatments tested (ANOVA), using Tukey’s procedure for determining homogeneous groups in STATISTICA 13.1. Averages marked with the same letter were not differentiated at the α = 0.05 significance level.

## 4. Conclusions

Under the conditions of deep fertilization, higher yields of potato tubers were obtained in comparison to surface fertilizer application. The optimum depth of fertilizer application was 20 cm. At that depth, the highest increase in yield was obtained in comparison to surface fertilizer application. In the conditions of deep application, the yields of the above-ground parts and root mass also increased. It should be emphasized, however, that the increase in the biomass of the above-ground parts and roots as a result of fertilization was considerably lower than the increase in the biomass of tubers, pointing to a considerably stronger response to fertilization of vegetative organs than storage organs. Very high fertilization of potatoes contributes considerably more to an increase in the biomass of above-ground parts and less to tuber yielding. Analysis of the uptake of nitrogen and phosphorus by potato plants and their distribution in the plant showed that deep application of fertilizers considerably increased the uptake of both nutrients in comparison to surface fertilization. The process of the accumulation of fertilizer nitrogen and phosphorus in tubers and vegetative parts, however, took a different course. In the above-ground parts and roots, with an increase in the level of fertilization, there was an increase in the accumulation of both nutrients. In tubers, the level of fertilization had practically no effect on the accumulation of either fertilizer nutrient, with a decreasing trend as the dose increased, as particularly manifested in reference to phosphorus. The advantage of deep over surface application was manifested not only in a higher yield of tubers and higher uptake of nutrients, nitrogen and phosphorus, but also in better management of nutrients by plants, reflected in respectively higher indices of use of nutrients from fertilizers.

Positive effects on the yielding of potatoes under the conditions of deep application in comparison to surface application were primarily caused by considerably better development of the root system, and particularly its larger surface area, substantially contributing to the potential for uptake of nutrients by plants.

## Figures and Tables

**Table 1:** Effect of depth and fertilization rate on total dry matter yield (g·plant^−1^).

Fertilizer Placement	Fertilizer Dosage			Average	Relative Value
	D_1_	D_2_	D_3_		
D0	67.6b ^1^	74.6b	84.7c	75.6b ^2^	100
D10	79.3b	86.6c	90.1c	85.3d	113
D20	83.5b	88.6b	93.1b	88.4d	117
D30	76.6b	81.1b	85.4b	81.0c	107
Average	76.8b	82.7c	88.3d	82.6	109
	D_0_ = 43.3a			43.3a	57

^1^ Average yield values for different fertilizer application dosages (in rows), denoted by the same letter, were not significantly differentiated at the level of α = 0.05. ^2^ Average yield values for different fertilizer placements (average—in column), denoted by the same letter, were not significantly differentiated at the level of α = 0.05.

**Table 2:** Effect of depth and fertilization rate on above-ground dry matter yield (g·plant^−1^).

Fertilizer Placement	Fertilizer Dosage			Average	Relative Value
	D_1_	D_2_	D_3_		
D0	17.6b ^1^	28.7c	42.0d	29.4c ^2^	100
D10	20.1b	34.7c	40.1c	31.7d	108
D20	22.7b	30.5c	37.8d	30.3cd	103
D30	20.5b	25.5c	34.6d	26.9b	91
Average	20.2b	29.9c	38.6d	29.6	100
	D_0_ = 7.7a			7.7a	26

^1^ Average yield values for different fertilizer application dosages (in rows), denoted by the same letter, were not significantly differentiated at the level of α = 0.05. ^2^ Average yield values for different fertilizer placements (average—in column), denoted by the same letter, were not significantly differentiated at the level of α = 0.05.

**Table 3:** Effect of depth and fertilization rate on tuber dry matter yield (g·plant^−1^).

Fertilizer Placement	Fertilizer Dosage			Average	Relative Value
	D_1_	D_2_	D_3_		
D0	44.8b ^1^	36.2a	30.2a	37.0a ^2^	100
D10	52.4b	43.3b	35.9a	43.8b	118
D20	54.6c	50.8bc	44.0b	49.8c	134
D30	49.7c	48.3bc	41.9b	46.6bc	126
Average	50.4d	44.6c	38.0b	44.3	120
	D_0_ = 33.0a			33.0a	89

^1^ Average yield values for different fertilizer application dosages (in rows), denoted by the same letter, were not significantly differentiated at the level of α = 0.05. ^2^ Average yield values for different fertilizer placements (average—in column), denoted by the same letter, were not significantly differentiated at the level of α = 0.05.

**Table 4:** Effect of depth and fertilization rate on root dry matter yield (g·plant^−1^).

Fertilizer Placement	Fertilizer Dosage			Average	Relative Value
	D_1_	D_2_	D_3_		
D0	5.5b ^1^	9.6c	12.4d	9.2cd ^2^	100
D10	6.4b	9.9c	13.9d	10.1d	110
D20	7.3b	7.8b	11.0c	8.7c	95
D30	5.9b	6.6bc	9.2c	7.2b	79
Average	6.3b	8.5c	11.6d	8.8	96
	D_0_ = 2.7a			2.7a	30

^1^ Average yield values for different fertilizer application rates (in rows), denoted by the same letter, were not significantly different at the level of α = 0.05. ^2^ Average yield values for different fertilizer placements (average—in column), denoted by the same letter, were not significantly differentiated at the level of α = 0.05.

**Table 5:** Effect of depth and fertilization rate on nitrogen and phosphorus uptake (g N, P plant^−1^) by dry weight of the whole plant.

Fertilizer Placement	Fertilizer Dosage						Average		Relative Value	
	D_1_		D_2_		D_3_		N	P	N	P
	N	P	N	P	N	P				
D0	1.51b ^1^	0.20b ^2^	1.86c	0.21bc	2.35d	0.23c	1.91b ^3^	0.21b ^4^	100	100
D10	1.77b	0.21b	2.31c	0.22b	2.65d	0.22b	2.24d	0.22bc	118	102
D20	1.85b	0.25b	2.40c	0.25b	2.68d	0.27b	2.31d	0.26d	121	120
D30	1.70b	0.23b	1.97b	0.23b	2.38c	0.24b	2.02c	0.23c	106	109
Average	1.71b	0.22b	2.14c	0.23bc	2.51d	0.24c	2.12	0.23	111	108
	D_0_ N = 0.66a						0.66a	0.13a	35	62
	D_0_ P = 0.13a									

^1,2^ Average values of N and P uptake for different fertilizer application rates (in rows), denoted by the same letter, were not significantly different at the level of α = 0.05. ^3,4^ Average values of N and P uptake for different fertilizer placements (average—in column), denoted by the same letter, were not significantly differentiated at the level of α = 0.05.

**Table 6:** Effect of depth and fertilization rate on whole-plant nitrogen and phosphorus uptake expressed as N:P ratio.

Fertilizer Placement	Fertilizer Dosage			Average	Relative Value
	D_1_	D_2_	D_3_		
D0	7.20b ^1^	8.86c	11.17c	9.08b ^2^	100
D10	7.89b	10.57c	12.16d	10.21c	112
D20	6.75b	9.27c	9.84c	8.62b	95
D30	6.29b	8.53c	9.96d	8.26b	91
Average	7.04b	9.31c	10.78d	9.04	100
	D_0_ = 5.16a			5.16a	57

^1^ Average N:P uptake values for different fertilizer rates (in rows), denoted by the same letter, were not significantly different at the level of α = 0.05. ^2^ Average N:P uptake values for different fertilizer placements (average—in column), denoted by the same letter, were not significantly differentiated at the level of α = 0.05.

**Table 7:** Chemical soil properties.

Parameter	Value	Level
pH (1M KCl)	5.8	Light acid
Available P (mg P_2_O_5_∙kg^−1^)	165.0	High
Available K (mg K_2_O∙kg^−1^)	145.0	Medium
Available Mg (mg MgO∙kg^−1^)	60.0	Medium
Clay content %	17.0	Light loamy sand

**Table 8:** Doses of nutrients in the pot experiment.

Nutrients		Fertilization Dosage			
		D_0_	D_1_	D_2_	D_3_
(g·pot^−1^)	N	0.0	1.30	2.60	3.90
	P_2_O_5_	0.0	0.65	1.30	1.95
	K_2_O	0.0	1.76	3.51	5.27
	NPK ^1^	0.0	3.71	7.41	11.12
	S—SO_4_	0.0	0.20	0.40	0.60

^1^ NPK—nutrient sum expressed as: N + P_2_O_5_ + K_2_O.

## Data Availability

The data are available by contacting the authors.
